# Risk classification for the transmission of vaccine-preventable diseases in the state of Minas Gerais, Brazil: 2018 to 2022

**DOI:** 10.1371/journal.pone.0311932

**Published:** 2024-12-05

**Authors:** Thales Philipe Rodrigues da Silva, Carolina Machado Moreira, Janaina Fonseca Almeida Souza, Eder Gatti Fernandes, Josianne Dias Gurmão, Ana Catarina de Melo Araújo, Aline Mendes Vimieiro, Fernanda Penido Matozinhos

**Affiliations:** 1 Department of Nursing in Women’s Health, School of Nursing Universidade Federal de São Paulo, São Paulo, Brazil; 2 Department of Maternal and Child Nursing and Public Health, Universidade Federal de Minas Gerais, Belo Horizonte, Minas Gerais, Brazil; 3 Superintendence of Epidemiological Surveillance, Secretaria de Estado de Saúde de Minas Gerais, Belo Horizonte, Brazil; 4 PhD in Collective Health from the Faculty of Medicine of the University of São Paulo–FMUSP, Department of Immunopreventable Diseases, Director of the National Immunization Program, Ministry of Health, Health and Environmental Surveillance Secretariat, São Paulo, Brazil; 5 Department of Immunopreventable Diseases, General Coordinator for Scientific Incorporation and Immunization, Ministry of Health, Secretariat for Health and Environmental Surveillance, General Coordinator for Scientific Incorporation and Immunization, PhD in Nursing from the State University of Pernambuco, Recife, Brazil; UFSJ: Universidade Federal de Sao Joao del-Rei, BRAZIL

## Abstract

The National Immunization Program (PNI) is one of Brazil’s most significant public health interventions. However, recent years have witnessed a progressive decline in vaccination coverage despite the success of the PNI and the expansion of Primary Health Care (PHC), the main point of entry for the population into health services. To address this challenge, broader strategies are needed, such as identifying areas at high risk for the transmission of vaccine-preventable diseases. This study aimed to analyze the risk classification for the transmission of vaccine-preventable diseases in the 853 municipalities of Minas Gerais, Brazil, from 2018 to 2022. This epidemiological time-series study uses secondary data on vaccination coverage, dropout rates, and homogeneity of the recommended immunobiologicals for children under 2 years of age from 2018 to 2022 in Minas Gerais. We obtained the data from the National Immunization Program Information System (SIPNI). The study highlighted a decline in vaccination coverage from 2018 to 2021, with a significant drop following the COVID-19 pandemic in 2020. According to the risk classification for the transmission of vaccine-preventable diseases, the proportion of municipalities classified as high and very high risk remained stable from 2018 to 2019, increased from 2019 to 2020 and from 2020 to 2021, and decreased from 2021 to 2022. We observed the public health impact not only regarding COVID-19 but also on most vaccine-preventable diseases. Given the scenario of declining vaccination coverage and the risk of a resurgence of vaccine-preventable diseases exacerbated by the COVID-19 pandemic, health services must implement public health strategies to mitigate this situation. Risk classification proved to be an effective methodology for prioritizing locations for health interventions. It enabled the analysis of the vaccination scenario in the state following the implementation of a participatory action research project conducted jointly by academia and health services.

## Introduction

Since the creation of Brazil’s National Immunization Program (PNI) in the 1970s and the expansion of the vaccination schedule for the Brazilian population, Brazil has been internationally recognized for its success in immunization efforts. The reduction in the incidence of vaccine-preventable diseases and, consequently, the reduction in deaths from these causes in Brazil were made possible because the PNI operates in a coordinated, hierarchical, and integrated manner. Over the years, this approach has allowed Brazil to achieve high vaccination coverage rates [1,2].

One of the goals set to ensure that countries reduce transmission, control, eliminate, and potentially eradicate vaccine-preventable diseases is a 95% vaccination coverage for most vaccines, according to the World Health Organization (WHO) [3]. In addition to the established coverage target, achieving homogeneous vaccination coverage across a territory is a crucial indicator of population safety [3]. High vaccination coverage is essential, but ensuring that coverage is homogeneously distributed at regional, municipal, and even neighborhood levels is equally necessary. Low homogeneity in vaccination coverage creates “pockets of susceptibility,” or areas with low coverage, poor uniformity, and a growing percentage of unprotected individuals. This can result in outbreaks of diseases that had been controlled for years or, in the case of newly introduced vaccines, hinder the achievement of recommended control targets, thereby putting the entire municipality, state, and country at risk [3].

Since 2015, Brazil has observed a decline in vaccination coverage in all 26 states and the Federal District [4–8], further exacerbated by the COVID-19 pandemic [9–11]. This scenario poses a serious public health problem, as it puts the population at risk for the transmission of vaccine-preventable diseases [12].

In Minas Gerais, which comprises 853 municipalities, a decline in vaccination coverage has also been observed, consequently increasing the risk of transmission of vaccine-preventable diseases. Studies conducted by the Observatory of Research and Studies in Vaccination (OPESV) at the School of Nursing, Federal University of Minas Gerais, in partnership with the Minas Gerais State Health Department (SES-MG), have highlighted this trend over the years [9,13–17]. The reduction in vaccination coverage is complex and multidimensional [18], with contextual, historical, sociocultural, environmental, and health system determinants, as well as economic, political, and individual factors interrelated and influencing immunization efforts [18]. Thus, using the risk classification methodology proposed by Braz et al. [19] has proven effective in identifying priority areas in Minas Gerais, Brazil, for developing strategies and actions to increase vaccination coverage [20].

This study aimed to analyze the risk classification for the transmission of vaccine-preventable diseases in the 853 municipalities of Minas Gerais, Brazil, from 2018 to 2022. Additionally, we sought to assess municipalities’ vulnerability to the occurrence of these diseases using vaccination data.

## Materials and methods

### Study design, population, and data collection

This study is an epidemiological, time-series analysis using secondary data on vaccination coverage, dropout rates, and homogeneity of recommended immunobiologicals for children under 2 years of age in Minas Gerais, Brazil, from 2018 to 2022.

Minas Gerais comprises 853 municipalities and has a territorial area of 586,522.122 km^2^. It is characterized by heterogeneity in income, education, and health conditions among municipalities. According to data from the Brazilian Institute of Geography and Statistics [21], the state has an estimated population of 20,539,989 people [21].

For the organization of health services, the state is divided into 19 Regional Health Superintendencies (SRS) and 9 Regional Health Management Units (GRS). According to Decree No. 47,769 of November 29, 2019, updated by Decree No. 48,661 of July 31, 2023, these units are responsible for implementing state health policies, advising on service organization, and coordinating, evaluating, and monitoring health actions.

### Variables’ description

The 853 municipalities in the state were classified based on their population size, as adapted from the reference framework of the Health Surveillance Actions Qualification Program (PQAVS) and previously presented in the study by Braz et al. [19]. The categories are as follows: small-sized (population less than or equal to 20,000 inhabitants), medium-sized (population between 20,001 and 100,000 inhabitants), and large-sized (population greater than or equal to 100,001 inhabitants) [19].

For this study, we analyzed the immunobiologicals described in Table 1. It is important to note that the measles-mumps-rubella (MMR) (second dose—D2) and varicella vaccines were included in the analysis starting in 2020, as vaccination coverage data for varicella became available in the information system that year.

**Table 1:** Immunobiologicals analyzed in the study according to the study years. Minas Gerais, Brazil, 2018 to 2022.

	Years				
Immunobiological	2018	2019	2020	2021	2022
Human rotavirus vaccine	X	X	X	X	X
Meningococcal C vaccine	X	X	X	X	X
Pneumococcal 10-valent vaccine	X	X	X	X	X
Pentavalent vaccine	X	X	X	X	X
Polio vaccine–IPV	X	X	X	X	X
Yellow fever vaccine	X	X	X	X	X
Measles-mumps-rubella vaccine (D1)	X	X	X	X	X
Measles-mumps-rubella vaccine (D2)				X	X
Hepatitis A vaccine	X	X	X	X	X
Varicella vaccine				X	X

**Notes**: Oral rotavirus vaccine: The second dose of the rotavirus vaccine in the public health system (SUS) and the second dose of the rota-pentavalent vaccine offered in the private sector were considered; meningococcal C vaccine: The second dose of meningococcal C and the second dose of meningococcal ACWY were considered; pentavalent vaccine: The third dose of the pentavalent vaccine and the third dose of the hexavalent vaccine in the private sector were considered; polio vaccine: The third dose of IPV, OPV, pentavalent in the private sector, and hexavalent in the private sector were considered; MMR (D1): The first dose of MMR, the first dose of quadrivalent, and the first dose of tetravalent were considered; MMR (D2): The second dose of MMR, the second dose of quadrivalent, and the second dose or single dose of tetravalent were considered; varicella vaccine: The first dose of varicella and the first dose of tetravalent were considered.

Vaccination coverage (VC) was calculated using the number of doses administered per immunobiological as the numerator and the population from the Live Birth Information System (SINASC) as the denominator for the following years: for 2018, the population from 2016; for 2019, the population from 2017; for 2020, the population from 2018; for 2021, the population from 2019; and for 2022, the population from 2020. Subsequently, vaccination coverage rates were categorized according to the goals set by the PNI, with adequate coverage defined as greater than or equal to 90% for the human rotavirus vaccine and greater than or equal to 95% for the other vaccines.

Regarding homogeneity among vaccines, we adopted the criteria proposed by Braz et al. [19] and agreed upon by SUS through the Public Health Action Organization Contract (COAP), as follows: adequate homogeneity (≥75% to 100% of vaccines with adequate coverage), low homogeneity (≥50% to <75% of vaccines with adequate coverage), and very low homogeneity (<50% of the analyzed vaccines with adequate vaccine coverage) [19]. The vaccination coverage homogeneity indicator is calculated as follows: the numerator is the number of vaccines analyzed that showed adequate coverage according to the PNI, divided by 8 until 2020 and by 10 from 2020 to 2022, and multiplied by 100.

Concerning the multi-dose vaccine dropout rate, we evaluated this indicator only for multi-dose vaccines, including the meningococcal C vaccine, pentavalent vaccine, pneumococcal 10 vaccine, polio vaccine, and oral human rotavirus vaccine. Dropout rates were classified as follows: low dropout rate (<5%), medium dropout rate (≥5% to ≤10%), and high dropout rate (≥10%) [19]. It should be noted that the pentavalent vaccine was excluded from the dropout rate calculation in 2019 due to immunobiological shortages in Minas Gerais.

We obtained all data from the Ministry of Health website (available at: <pni.datasus.gov.br>).

Finally, for the five-year analysis period (2018 to 2022), we classified the municipalities of Minas Gerais according to their risk of transmission of vaccine-preventable diseases into five categories [19]:

- Very low—Municipality with Vaccination Coverage Homogeneity (VCH) = 100%.
- Low—Municipality with VCH ≥75% and <100%, with adequate coverage for polio, MMR (international disease elimination commitment), and pentavalent vaccine, which is considered the “standard marker” for the quality of vaccination services (three-dose injectable scheme);
- Medium—municipality with VCH ≥75% and <100% and vaccine coverage below the goal for one or more of the following vaccines: polio, measles-mumps-rubella, or pentavalent;
- High—Municipality with VCH <75%, regardless of vaccination coverage;
- Very high–Municipality with VCH <75%, high dropout rate (≥10%) for any of the evaluated vaccines, large population size, and municipalities with no vaccination records for any vaccine, regardless of population size.

It is noteworthy that in 2018 and 2019, none of the municipalities had missing dose records. In 2020, 2 (0.23%) municipalities did not register any vaccines, with 1 municipality registering for 3 vaccines (human rotavirus vaccine, pneumococcal vaccine, and pentavalent vaccine) and the other not registering for the varicella vaccine. In 2021, 10 (1.17%) municipalities were absent from the register: 1 municipality was absent from registration for 5 vaccines (human rotavirus vaccine, meningococcal C vaccine, pneumococcal vaccine, pentavalent vaccine, and polio vaccine), 1 municipality was absent from registration for 3 vaccines (human rotavirus vaccine, meningococcal C vaccine, and pneumococcal vaccine), and 8 municipalities were absent from registration for the second dose of the MMR vaccine. Finally, in 2022, 2 (0.23%) municipalities did not register for any of the vaccines, one of which did not register for the meningococcal C vaccine, and the other did not register for the second dose of the MMR vaccine.

After classifying the municipalities, we observed a small number classified as medium and very high risk, so these categories were grouped as follows: low with medium risk and high with very high risk of transmission of vaccine-preventable diseases through immunization efforts.

### Statistical analysis

We analyzed data using Statistical Software for Professional (Stata), version 16.0. We compared the data over the years (2018 to 2022) using the McNemar test for proportions of the multi-dose vaccine dropout rate, vaccination coverage homogeneity (VCH), and risk classification for the transmission of vaccine-preventable diseases. The Conover test with Bonferroni correction was used for multiple comparisons between each pair of years to detect where differences between classifications occurred. We adopted the significance level of 5% for all analytical procedures.

#### Ethics approval and consent to participate in the study

This study utilized publicly available data that does not allow for individual identification; therefore, ethical approval from a Research Ethics Committee was not required.

## Results

Table 2 presents the percentage of municipalities according to the classification of vaccination coverage for the years analyzed. For all vaccines evaluated, there was a decrease in the percentage of municipalities classified as having adequate vaccination coverage between 2018 and 2021. However, in 2022, there was an increase in the percentage of municipalities classified as having adequate vaccination coverage for all vaccines. Notably, for the Hepatitis A vaccine, the percentage of municipalities classified as having adequate vaccination coverage in 2022 was closer to that of 2018 (a difference of 11.96%) (Table 2).

**Table 2:** Percentage of municipalities according to vaccination coverage classification, Minas Gerais, Brazil, 2018 to 2022.

	Years				
	2018	2019	2020	2021	2022
	n(%)	n(%)	n(%)	n(%)	n(%)
**Vaccination coverage**					
**Oral Human Rotavirus Vaccine**					
Adequate (≥ the goal)	542(63.54)	282(34.90)	268(31.53)	166(19.71)	407(47.71)
Low (≥ 50% and less than the goal)	221(25.91)	312(38.61)	389(45.76)	422(50.12)	357(41.85)
Very low (0% to <50%)	90(10.55)	214(26.49)	193(22.71)	254(30.17)	89(10.43)
**Pneumococcal vaccine**					
Adequate (≥ the goal)	605(70.93)	495(58.03)	434(50.94)	264(31.02)	447(52.40)
Low (≥ 50% and less than the goal)	243(28.49)	351(41.15)	379(44.48)	518(60.87)	398(46.66)
Very low (0% to <50%)	5(0.59)	7(0.82)	39(4.58)	69(8.11)	8(0.94)
**Meningococcal C vaccine**					
Adequate (≥ the goal)	566(66.35)	467(54.75)	408(47.89)	241(29.32)	381(44.67)
Low (≥ 50% and less than the goal)	277(32.47)	376(44.08)	401(47.07)	537(63.10)	461(54.04)
Very low (0% to <50%)	10(1.17)	10(1.17)	43(5.05)	73(8.58)	11(1.29)
**Pentavalent vaccine**					
Adequate (≥ the goal)	567(66.47)	277(32.47)	446(52.35)	266(31.22)	411(48.18)
Low (≥ 50% and less than the goal)	281(32.94)	552(64.71)	360(42.25)	511(59.98)	431(50.53)
Very low (0% to <50%)	5(0.59)	24(2.81)	46(5.40)	75(8.80)	11(1.29)
**Polio vaccine**					
Adequate (≥ the goal)	561(65.77)	461(54.04)	413(48.42)	245(28.76)	412(48.30)
Low (≥ 50% and less than the goal)	286(33.53)	383(44.90)	403(47.25)	528(61.97)	429(50.29)
Very low (0% to <50%)	6(0.70)	9(1.06)	37(4.34)	79(9.27)	12(1.41)
**Measles-Mumps-Rubella (D1)**					
Adequate (≥ the goal)	575(67.41)	597(69.99)	505(59.20)	323(37.87)	444(52.05)
Low (≥ 50% and less than the goal)	271(31.77)	251(29.43)	314(36.81)	460(53.93)	396(46.42)
Very low (0% to <50%)	7(0.82)	5(0.59)	34(3.99)	70(8.21)	13(1.52)
**Yellow Fever vaccine**					
Adequate (≥ the goal)	485(56.86)	410(48.07)	345(40.45)	215(25.21)	261(30.60)
Low (≥ 50% and less than the goal)	363(42.56)	432(50.64)	464(54.40)	550(64.48)	570(66.82)
Very low (0% to <50%)	5(0.59)	11(1.29)	44(5.16)	88(10.32)	22(2.58)
**Hepatitis A vaccine**					
Adequate (≥ the goal)	456(53.46)	501(59.73)	453(53.11)	252(29.54)	354(41.50)
Low (≥ 50% and less than the goal)	387(45.37)	340(39.86)	354(41.50)	518(60.73)	484(56.74)
Very low (0% to <50%)	10(1.17)	12(1.41)	46(5.39)	83(9.73)	15(1.76)

For the multi-dose vaccine dropout rate, we observed a statistically significant increase (p<0.05) in the proportion of municipalities classified as having a “low” multi-dose vaccine dropout rate between 2018 and 2022. The multi-dose vaccine dropout rate for the oral human rotavirus vaccine increased from 2018 to 2019 in the low dropout rate classification. From 2019 to 2021, the rate remained stable, followed by an increase between 2021 and 2022 in the low multi-dose vaccine dropout rate classification, with a statistically significant difference (p<0.05).

As for the multi-dose vaccine dropout rate for the Pneumo10 and Pneumo13 vaccines, the pattern was stable in consecutive pairs of years. However, we observed a statistically significant increase (p<0.05) in municipalities classified as having a low multi-dose vaccine dropout rate. Regarding the multi-dose vaccine dropout rate for the pentavalent and hexavalent vaccines, we found a reduction from 2018 to 2019 in municipalities classified as having a low multi-dose vaccine dropout rate, followed by an increase from 2019 to 2020 and again from 2020 to 2021. However, there was a reduction between 2021 and 2022 in the multi-dose vaccine dropout rate classified as low, with all results showing a statistically significant difference (p<0.05).

For the classification of “adequate” vaccination coverage homogeneity (≥ 75% to ≤ 100%), there was a reduction between 2018 and 2022 (p<0.05) (Table 3). From 2018 to 2021, we observed a decrease in vaccination coverage homogeneity among municipalities classified as having adequate homogeneity (≥75% to 100%), reaching its lowest point in 2021. However, an increase was observed from 2021 to 2022, with a statistically significant difference (p<0.05).

**Table 3:** Multi-dose vaccine dropout rate, vaccination coverage homogeneity, and risk classification for the transmission of vaccine-preventable diseases, Minas Gerais, Brazil, 2018 to 2022.

	Years					p-value
	2018	2019	2020	2021	2022	
	n(%)	n(%)	n(%)	n(%)	n(%)	
**Multi-dose vaccine abandonment rate**						
**Oral Human Rotavirus Vaccine**						**<0.005** *
	A	B	BC	B	C	
Low (<5%)	543(63.66)	631(73.97)	665(78.14)	628(73.80)	700(82.26)	
Medium (≥5% to ≤10%)	174(20.40)	119(13.95)	97(11.40)	112(13.16)	92(10.79)	
High (>10%)	136(15.94)	103(12.08)	89(10.46)	111(13.04)	61(7.15)	
**Pneumo10 and 13**	A	AB	B	AB	B	**<0.005** *
Low (<5%)	579(67.88)	628(73.62)	663(77.91)	623(73.21)	642(75.26)	
Medium (≥5% to ≤10%)	166(19.46)	131(15.36)	98(11.52)	111(13.04)	123(14.42)	
High (>10%)	108(12.66)	94(11.02)	90(10.58)	117(13.75)	88(10.32)	
**Pentavalent and Hexavalent** A		B	A	C	A	**<0.005** *
Low (<5%)	460(53.93)	342(40.09)	484(56.94)	578(67.84)	505(59.20)	
Medium (≥5% to ≤10%)	168(19.70)	120(14.07)	127(14.94)	95(11.15)	154(18.05)	
High (>10%)	225(26.38)	391(45.84)	239(28.12)	179(21.01)	194(22.74)	
**VIP, VOP, Hexavalent, and Pentavalent** A AB			B	B	AB	**<0.005** *
Low (<5%)	458(53.69)	511(59.91)	534(62.68)	533(62.56)	516(60.49)	
Medium (≥5% to ≤10%)	152(17.82)	146(17.12)	143(16.78)	115(13.50)	139(16.30)	
High (>10%)	243(28.49)	196(22.98)	175(20.54)	204(23.94)	198(23.21)	
Vaccination Coverage Homogeneity** A B			C	D	C	<0.005*
≥75% to 100%	432(50.64)	395(46.31)	312(36.58)	163(19.11)	261(30.60)	
≥50% to <75%	164(19.23)	126(14.77)	139(16.30)	89(10.43)	167(19.58)	
≥0% to <50%	257(30.13)	332(38.92)	402(47.13)	601(70.46)	425(49.82)	
**Risk Classification** A		A	B	C	D	<0.005*
Very Low	162(18.99)	303(35.52)	164(19.23)	80(9.38)	115(13.48)	
Low and Medium	270(31.65)	92(10.79)	148(17.35)	83(9.73)	173(20.28)	
High and Very High	421(49.36)	458(53.69)	541(63.42)	690(80.89)	565(66.23)	

Note

*McNemar test comparing 2018 with 2019, 2019 with 2020, 2020 with 2021, 2021 with 2022, and 2018 with 2022. Identical letters indicate similarity between the proportions of municipalities, i.e., no statistical difference in the presented comparison.

** Adequate according to the COAP criteria.

Regarding the classification of risk for the transmission of vaccine-preventable diseases, in 2018, 49.36% of the municipalities in Minas Gerais were classified as high and very high risk. By 2022, this percentage increased to 69.44% (p<0.05). The proportion of municipalities classified as high and very high risk, according to the risk classification for the transmission of vaccine-preventable diseases, remained stable from 2018 to 2019, increased from 2019 to 2020 and from 2020 to 2021, and decreased from 2021 to 2022, with a statistically significant difference (p<0.05) (Table 3).

## Discussion

This study demonstrated a decline in vaccination coverage from 2018 to 2021, with a significant drop observed following the pandemic period in 2020, highlighting the impact on public health not only related to COVID-19 but also to most vaccine-preventable diseases. The COVID-19 pandemic imposed a substantial burden on health services [22]. Although recommendations were made to maintain immunization activities, researchers have shown a significant decrease in vaccination coverage among children [23–26].

Maintaining routine vaccination during the pandemic posed a challenge, as people were hesitant to visit healthcare facilities, and many locations were not prepared to manage social distancing and other virus transmission control measures [4]. Balancing protection against diseases that continued to circulate among the population became a significant challenge. Another factor that undoubtedly affected herd immunity was the spread of misinformation (fake news), particularly during a period of societal uncertainty, thereby leading to vaccine hesitancy and putting millions of people at risk [27].

The decline in vaccination rates in recent years, particularly among children, is concerning. In 2016, Brazil received certification from the Pan American Health Organization (PAHO) as a region free of measles virus circulation. However, significant declines in vaccination coverage rates for the measles-mumps-rubella (MMR) vaccine have since been observed in the country [28,29].

In response to these concerns, since 2021, the OPESV, in partnership with the SES-MG, initiated a project titled “Strategies to Increase Vaccination Coverage among Children under 2 Years of Age in Minas Gerais, Brazil: An Action Research Approach.” The project involved targeted and coordinated actions to improve this scenario [20]. Workshops were conducted with each priority regional health unit (identified in previous studies) [13,17] to better understand the weaknesses and successes across municipalities, setting goals and developing plans for change and reinforcement [20].

Through this collaborative research between academia and health services, we contribute to public policies to improve municipalities’ vaccination coverage and risk classification. The project implemented several strategies, such as enhancing community engagement, extending health post hours, providing team training, conducting outreach to those who missed vaccinations, verifying vaccination cards, organizing off-site campaigns, and forming partnerships with sectors of education, leisure, and culture, using tailored strategies for specific target audiences [30,31]. This action research demonstrated the commitment of healthcare professionals who implemented the discussed planning in workshops, which were fundamental for these efforts’ continuity, maintenance, and sustainability, ensuring ongoing success [32].

We emphasize the importance of Primary Health Care in promoting health and achieving good vaccination coverage, as a more comprehensive and attentive approach allows for more effective surveillance tailored to the specificities of each territory [33]. The relationship with citizens, underscored in this action research, fosters trust and facilitates better organization and execution of health actions, ensuring strategies that more effectively promote societal adherence to immunization.

Another point worth highlighting is the role of the Family Health Strategy (ESF) developed within the scope of Primary Health Care. The ESF is a crucial policy in ensuring equitable care, reducing infant mortality [34], and increasing vaccination coverage in the state [13]. In recent years, Minas Gerais has seen an increase in the proportion of people living in households registered with these units, from 72.3% in 2013 to 73.0% in 2019 [35]. While the expansion of the FHS is a positive factor in Minas Gerais, it remains challenging due to the state’s large number of municipalities and diverse contexts [13].

This study also revealed progress in the vaccination coverage homogeneity among municipalities and a reduction in the multi-dose vaccine dropout rate. This progress may be associated with increased awareness and sensitivity, indicating that the effort to regain high vaccination rates in the country involves a combination of factors [30].

Reiterating strategies to promote vaccination adherence within the territory is crucial. This will ensure adequate and homogeneous vaccination coverage and, consequently, reduce the risk classification [2,36].

Finally, regarding limitations, although this study used secondary data, which may lead to underestimation due to potential data entry errors in the SI-PNI system, it presents important discussion points regarding vaccination coverage surveillance in Minas Gerais and, consequently, Brazil. Another limitation worth mentioning is that, according to the risk classification for vaccine-preventable diseases proposed by Braz et al. [19], municipalities with no records for any analyzed vaccine doses were automatically classified as at very high risk for vaccine-preventable disease transmission. Therefore, for administrative and/or management reasons, such municipalities could theoretically be at very low risk.

## Conclusions

Given the scenario of declining vaccination coverage and the risk of a resurgence of vaccine-preventable diseases exacerbated by the COVID-19 pandemic, health services must implement public health strategies to address this situation.

Risk classification proved to be an effective methodology for prioritizing locations for intervention and analyzing the vaccination scenario in the state following the implementation of a participatory action research project carried out jointly between academia and health services.
